# Clearance of archived integrase strand transfer inhibitors resistance mutations in people with virologically suppressed HIV infection

**DOI:** 10.1093/jacamr/dlae194

**Published:** 2024-12-05

**Authors:** Basma Abdi, Romain Palich, Sophie Seang, Antoine Fauchois, Théophile Cocherie, Antoine Faycal, Sophie Sayon, Elisa Teyssou, Sanaa Saliba, Cathia Soulie, Marc Antoine Valantin, Valérie Pourcher, Christine Katlama, Vincent Calvez, Anne-Geneviève Marcelin, Marc Wirden

**Affiliations:** Sorbonne Université, INSERM, Institut Pierre Louis d’Epidémiologie et de Santé Publique (IPLESP UMRS 1136), AP-HP, Hôpital Pitié Salpêtrière, Laboratoire de Virologie, Paris, France; Sorbonne Université, INSERM, Institut Pierre Louis d’Epidémiologie et de Santé Publique (IPLESP UMRS 1136), AP-HP, Hôpital Pitié Salpêtrière, Service des Maladies Infectieuses et Tropicales, Paris, France; Sorbonne Université, INSERM, Institut Pierre Louis d’Epidémiologie et de Santé Publique (IPLESP UMRS 1136), AP-HP, Hôpital Pitié Salpêtrière, Service des Maladies Infectieuses et Tropicales, Paris, France; Sorbonne Université, INSERM, Institut Pierre Louis d’Epidémiologie et de Santé Publique (IPLESP UMRS 1136), AP-HP, Hôpital Pitié Salpêtrière, Laboratoire de Virologie, Paris, France; Sorbonne Université, INSERM, Institut Pierre Louis d’Epidémiologie et de Santé Publique (IPLESP UMRS 1136), AP-HP, Hôpital Pitié Salpêtrière, Laboratoire de Virologie, Paris, France; Sorbonne Université, INSERM, Institut Pierre Louis d’Epidémiologie et de Santé Publique (IPLESP UMRS 1136), AP-HP, Hôpital Pitié Salpêtrière, Service des Maladies Infectieuses et Tropicales, Paris, France; Sorbonne Université, INSERM, Institut Pierre Louis d’Epidémiologie et de Santé Publique (IPLESP UMRS 1136), AP-HP, Hôpital Pitié Salpêtrière, Laboratoire de Virologie, Paris, France; Sorbonne Université, INSERM, Institut Pierre Louis d’Epidémiologie et de Santé Publique (IPLESP UMRS 1136), AP-HP, Hôpital Pitié Salpêtrière, Laboratoire de Virologie, Paris, France; Sorbonne Université, INSERM, Institut Pierre Louis d’Epidémiologie et de Santé Publique (IPLESP UMRS 1136), AP-HP, Hôpital Pitié Salpêtrière, Service des Maladies Infectieuses et Tropicales, Paris, France; Sorbonne Université, INSERM, Institut Pierre Louis d’Epidémiologie et de Santé Publique (IPLESP UMRS 1136), AP-HP, Hôpital Pitié Salpêtrière, Laboratoire de Virologie, Paris, France; Sorbonne Université, INSERM, Institut Pierre Louis d’Epidémiologie et de Santé Publique (IPLESP UMRS 1136), AP-HP, Hôpital Pitié Salpêtrière, Service des Maladies Infectieuses et Tropicales, Paris, France; Sorbonne Université, INSERM, Institut Pierre Louis d’Epidémiologie et de Santé Publique (IPLESP UMRS 1136), AP-HP, Hôpital Pitié Salpêtrière, Service des Maladies Infectieuses et Tropicales, Paris, France; Sorbonne Université, INSERM, Institut Pierre Louis d’Epidémiologie et de Santé Publique (IPLESP UMRS 1136), AP-HP, Hôpital Pitié Salpêtrière, Service des Maladies Infectieuses et Tropicales, Paris, France; Sorbonne Université, INSERM, Institut Pierre Louis d’Epidémiologie et de Santé Publique (IPLESP UMRS 1136), AP-HP, Hôpital Pitié Salpêtrière, Laboratoire de Virologie, Paris, France; Sorbonne Université, INSERM, Institut Pierre Louis d’Epidémiologie et de Santé Publique (IPLESP UMRS 1136), AP-HP, Hôpital Pitié Salpêtrière, Laboratoire de Virologie, Paris, France; Sorbonne Université, INSERM, Institut Pierre Louis d’Epidémiologie et de Santé Publique (IPLESP UMRS 1136), AP-HP, Hôpital Pitié Salpêtrière, Laboratoire de Virologie, Paris, France

## Abstract

**Introduction:**

We assessed the kinetics of the clearance of integrase strand transfer inhibitors resistance mutations (INSTIs-RMs) and associated factors from people living with HIV (PWH) displaying suppressed viral replication after virological failure (VF) on an INSTI regimen.

**Patients and methods:**

We included PWH with HIV-RNA viral loads ≤20 copies/mL for at least 5 years in whom INSTIs-RM had been identified at least once in a prior RNA resistance genotyping test. HIV DNAs were sequenced by Sanger sequencing (SS) and ultra-deep sequencing (UDS; detection threshold: 5%) every year over the preceding 5 years.

**Results:**

We included 39 PWH in the study. Most (95%) had experienced VF on a raltegravir-containing regimen. The past INSTIs-RMs were not detected in the peripheral blood mononuclear cells of 35 of the 39 (90%) PWH by SS at the end of follow-up. In a longitudinal analysis (2017–21) based on UDS, the previously detected INSTIs-RMs were not detected in 29 of the 35 (83%) PWH. In multivariable analysis, the duration of viral replication and the level of HIV-RNA during prior VF were significantly associated with the persistence of INSTIs-RM, with odds ratios of 1.05 per week of replication (95% CI, 1.00–1.11; *P *= 0.024) and 8.26 per log_10_ copies/mL (95% CI, 1.46–46.59; *P *= 0.017).

**Conclusions:**

We observed a clear trend towards the clearance of archived INSTIs-RM after a long period of virological control leading to changes in the resistance profile in cellular DNA, raising the possibility of studies assessing the recycling of INSTI classes even in the presence of a history of resistance.

## Background

Integrase strand transfer inhibitors (INSTIs) are a major class of ART used to treat people living with HIV (PWH).^1–5^ They have several key benefits including the very effective suppression of HIV replication, rapidly reducing viral load and preserving immune function.^3,6^ INSTIs act rapidly by blocking the integrase (INT), a key enzyme in the HIV replication cycle, leading to a rapid decrease in viral load.^6^ INSTIs are generally well tolerated and have a favourable safety profile, often associated with fewer side effects than other classes of antiretroviral drugs.^7^ Since their initial identification as promising candidate therapeutic drugs, five INSTIs have been approved by the US Food and Drug Administration: raltegravir (RAL), elvitegravir (EVG), dolutegravir (DTG), bictegravir (BIC) and cabotegravir (CAB). With their high genetic barrier, INSTIs are becoming the recommended first choice in HIV guidelines for both antiretroviral drug-naive and previously treated PWH, and CAB has become the leading long-acting agent for HIV-1 treatment.^8–11^

Resistance rapidly developed following virological failure (VF) on first-generation INSTIs (RAL and EVG), increasing the need for new improved INSTIs with little or no cross-resistance.^12–17^ Most PWH experiencing VF on a RAL- or EVG-containing regimen have been shown to carry not only viruses with mutations conferring a high degree of resistance to RAL and EVG but also cross-resistance to DTG, BIC and CAB.^2,13,18–22^ One of the principal limitations of these second-generation INSTIs for use in ART is this potential cross-resistance due to mutations selected in the past on RAL- and EVG-containing regimens.

ART guidelines do not recommend the use of drugs for which resistance mutations (RMs) are known to have developed, as this is a known risk factor for VF.^23,24^ Circulating drug-resistant HIV-1 variants can be archived in viral reservoirs, where they can persist for unknown durations, re-emerging in the presence of therapeutic selective pressure.^25,26^ These archived variants with drug resistance can be explored by sequencing HIV sequences in DNA from the cells of PWH with HIV-RNA levels below the limit of detection or with low-level viraemia.^27^ Several studies have suggested that RM may be cleared over time in PWH in whom viral replication is controlled.^28–30^

Given the increasing interest in the use of second-generation INSTIs for HIV-1 treatment and the inclusion of these drugs in all recommended antiretroviral regimens, together with long-acting HIV pre-exposure prophylaxis (PrEP), the possibility of recycling molecules for which there is a history of resistance has become a major issue. We therefore investigated the kinetics of INSTIs-RM in peripheral blood mononuclear cells (PBMCs) and the factors associated with their clearance in PWH with suppressed HIV-1 replication and prior VF on an INSTI-based regimen.

## Materials and methods

### Study design and patients

We retrospectively studied PWH followed at the Department of Infectious Diseases of Pitié-Salpêtrière Hospital (Paris, France) between 2017 and 2021. We included all PWH with an HIV-RNA viral load ≤20 copies/mL for at least 5 years in whom INSTIs-RM had been identified at least once in a previous RNA genotype resistance test.

The characteristics (sex and age) of the participants and their medical history of HIV infection [time since HIV diagnosis, time on ART, time to undetectable HIV-RNA, zenith HIV-RNA levels, HIV subtype, CD4 nadir, last CD4 count, duration of viral replication on INSTIs, mean viral load (number of HIV-RNA copies) during viral replication on INSTIs, time since the last detection of INSTIs-RM and ongoing ART] were recorded at inclusion, for the timepoint at which the last available blood sample was collected.

### Sanger and ultra-deep sequencing

Analyses were performed retrospectively on whole blood collected over a period of 5 years and frozen at −20°C. For all PWH, we used Sanger sequencing (SS) to sequence the INT region of HIV in cellular DNA from the PBMCs in the most recently collected sample available in 2021. The French National Agency for Research on AIDS and Emerging Infectious Diseases (ANRS-MIE) protocol was followed.

If INSTIs-RMs were not detected by SS in the most recent sample in 2021, we performed ultra-deep sequencing (UDS) on five samples from the PWH (one sample per year between 2017 and 2021). UDS was performed with Illumina technology (Illumina, San Diego, CA, USA). We deep-sequenced two fragments of the INT region: INT1 and INT2. The primers and polymerase chain reaction procedures used for UDS are detailed in the Supplemental Data (available as Supplementary data at *JAC-AMR* Online). Library purification/quantification and sequencing were performed in accordance with the manufacturer’s recommendations. Sequences were demultiplexed automatically on the MiSeq platform during the data-processing steps, and two paired fastq files were generated for each sample, representing the two paired-end reads. For the identification of INSTIs-RM, the sequence reads were analysed with IDNS^®^  ^©^ SmartGene 2019 (an advanced sequencing platform), and resistance (cut-off for the detection of minority resistant variants for UDS sequences = 5%) was interpreted with the latest ANRS-MIE resistance algorithm (October 2022, V33). Variants present in more than 20% of the quasi-species were considered to be major resistant variants. Variants present at a proportion between 5% and 20% were considered to be minority variants (MRVs).

### Statistical analysis

All the reported values are medians with the IQR for continuous variables and frequencies and percentages for categorical variables. We defined two groups of participants according to INSTIs-RM detection in DNA from PBMCs during the study period (2017–21): the INSTIs-RM^+^ group (INSTIs-RM detected by SS or UDS at least once during the study period) and the INSTIs-RM^−^ group (no INSTIs-RM detected by SS or UDS during the study period).

We used Mann–Whitney *U*-tests and the Chi2 tests to compare data between the two groups of participants. Univariable and multivariable models were used to identify immunological and virological factors associated with the persistence of INSTIs-RM over time. Variables with a *P* value <0.2 in univariable analysis were retained in the multivariable analysis. All reported *P* values are two tailed, with a significance threshold of *P* = 0.05. Analyses were performed with SPSS statistics version 23.0 for Windows.

### Ethics

This retrospective study was conducted in accordance with good clinical practice, the ethical principles of the Helsinki declaration and ANRS standard practices for clinical research. Data were collected with the NADIS computerized medical chart, for which all PWH provided signed consent for use of the information recorded. All data were anonymized before analysis. PWH were systematically notified of any supplementary biological analyses on frozen samples initially collected as part of routine clinical practice.

## Results

### Study population

**Figure 1.**
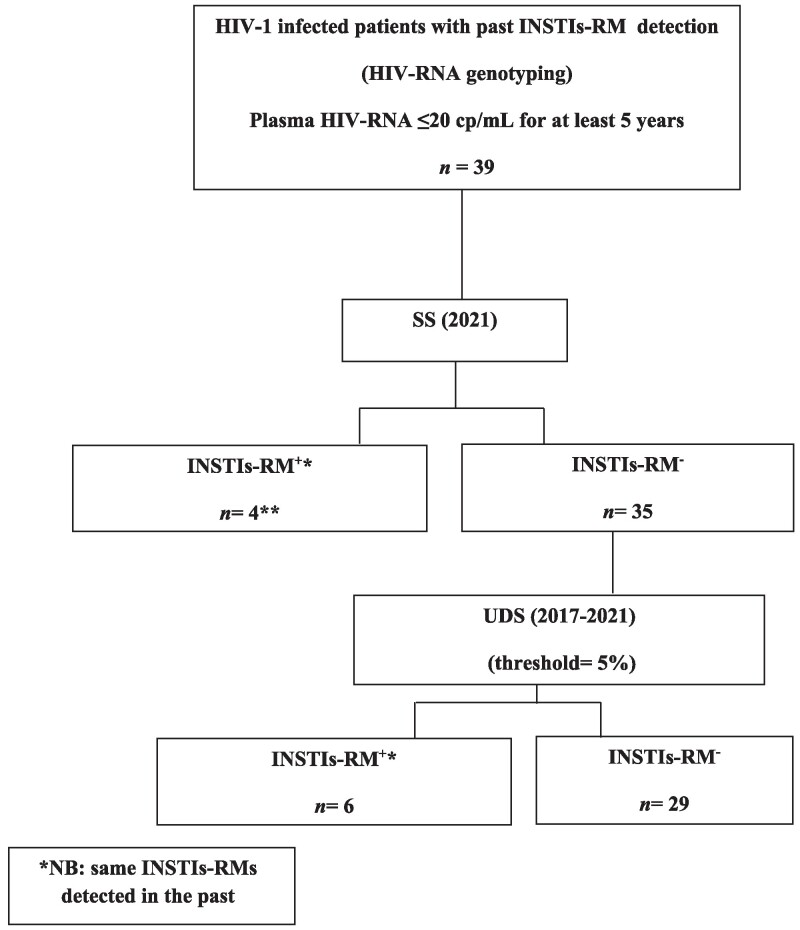
Study flowchart. INSTIs-RM, mutation conferring resistance to one or more INSTIs; INSTIs-RM^+^, INSTIs-RM detected by SS or as MRV ≥5% by UDS; INSTIs-RM^−^, no detection of INSTIs-RM by SS and UDS. **Four PWH had previous UDS results available from earlier samples.

We included 39 PWH with strict viral control (HIV viral load always ≤20 copies/mL over the last 5 years) for whom INSTIs-RM had been identified at least once in the past, in a previous RNA genotype resistance test (Figure 1).

All these PWH had experienced prior VF on ART containing RAL (94.87%) or EVG (5.12%). The median duration of viral replication on these INSTIs was 13.14 weeks (5.00–27.17), with a median HIV-RNA level of 3.40 (2.76–3.74) log_10_ cp/mL. The median time between the last detection of INSTIs-RM in RNA and inclusion in 2021 was 9 years (IQR: 7–12). The following INSTIs-RM had been detected in the past: N155H (*n *= 25), N155H + Q148K/R (*n *= 3), Q148K/R + G140C/S (*n *= 8), Q148K/R (*n *= 2) and Q148K/R + E138K (*n *= 1).

The characteristics of the participants are shown in Table 1. Most were men (74.35%), and the median age was 58 years (IQR: 55–65 years). Their HIV-1 infection was diagnosed a median of 30 years ago (IQR: 25–35 years); they had been on ART for a median of 25 years (IQR: 21–28 years) and had an HIV-RNA viral load ≤ 20 copies/mL for a median of 7.38 years (IQR: 6.09–10.48 years). The participants were mostly infected with B-subtype viruses (94.88%) and their current ART included INSTIs in 48.72% of cases.

**Table 1.** Patient characteristics

Characteristics	All patients *N* = 39	INSTIs-RM^−^ *N *= 29	INSTIs-RM^+^ *N* = 10	*P* value
Sex, *n* (%)				
Male	29 (74.35)	21 (72.42)	8 (80)	0.636
Female	10 (25.64)	8 (27.58)	2 (20)	
Age, years, median (IQR)	58 (55–65)	58 (55–65)	55 (53–62)	0.221
Time since HIV diagnosis, years, median (IQR)	30 (25–35)	32 (26–35)	28 (20–35)	0.687
Time on ART, years, median (IQR)	25 (21–28)	26 (25–29)	23 (19–26)	0.100
Time to undetectable HIV-RNA, years, median (IQR)	7.38 (6.09–10.48)	7.38 (6.17–10.55)	7.30 (5.84–9.88)	0.872
Zenith HIV-RNA viral load, log_10_ copies/mL, median (IQR)	5.20 (4.32–5.64)	5.07 (3.99–5.49)	5.56 (5.08–6.00)	**0**.**038**
HIV subtype, *n* (%)				
B	37.00 (94.88)	27.00 (93.10)	10.00 (100.00)	0.695
Non-B^a^	2.00 (5.12)	2.00 (6.90)	0.00 (0.00)	
CD4 nadir, cells/mm^3^, median (IQR)	128 (39–230)	147 (78–235)	56 (6–138)	0.097
Last count of CD4 cells/mm^3^, median (IQR)	569.00 (315–789)	642 (399–821)	403 (238–616)	**0**.**036**
Past INSTIs				
EVG	2.00 (5.12)	2.00 (6.90)	0.00 (0.00)	0.394
RAL	37.00 (94.87)	27.00 (93.10)	10.00 (100.00)	
Duration of viral replication on INSTIs, weeks, median (IQR)	13.14 (5.00–27.17)	10.57 (3.42–22.14)	32 (6.30–71.43)	**0**.**038**
Mean HIV-RNA during viral replication on INSTIs, log_10_ copies/mL, median (IQR)	3.40 (2.76–3.74)	2.90 (2.67–3.55)	4.11 (3.40–5.10)	**0**.**007**
Time since last detection of INSTI-RMs, years, median (IQR)	9.00 (7.00–12.00)	10 (7.00–12.00)	8.00 (7.00–12.00)	0.584
INSTIs included in current ART, *n* (%)	19.00 (48.72)	13.00 (44.82)	6.00 (60.00)	0.408

INSTIs-RM^+^, INSTIs-RM detected by SS or UDS (MRV ≥ 5%); INSTIs-RM^−^, no INSTIs-RM detected by SS or UDS.

All the reported values are medians with the IQR for continuous variables and frequencies and percentages for categorical variables. Mann–Whitney and Chi^2^ tests were used for comparisons between INSTIs-RM^+^ and INSTIs-RM^−^ patients. Statistical significance (*P *< 0.05) is indicated by *P* values in bold.

^a^HIV subtype non-B = CRF02 _AG (1), D (1).

### INSTIs-RM detection by SS and UDS

**Figure 2.**
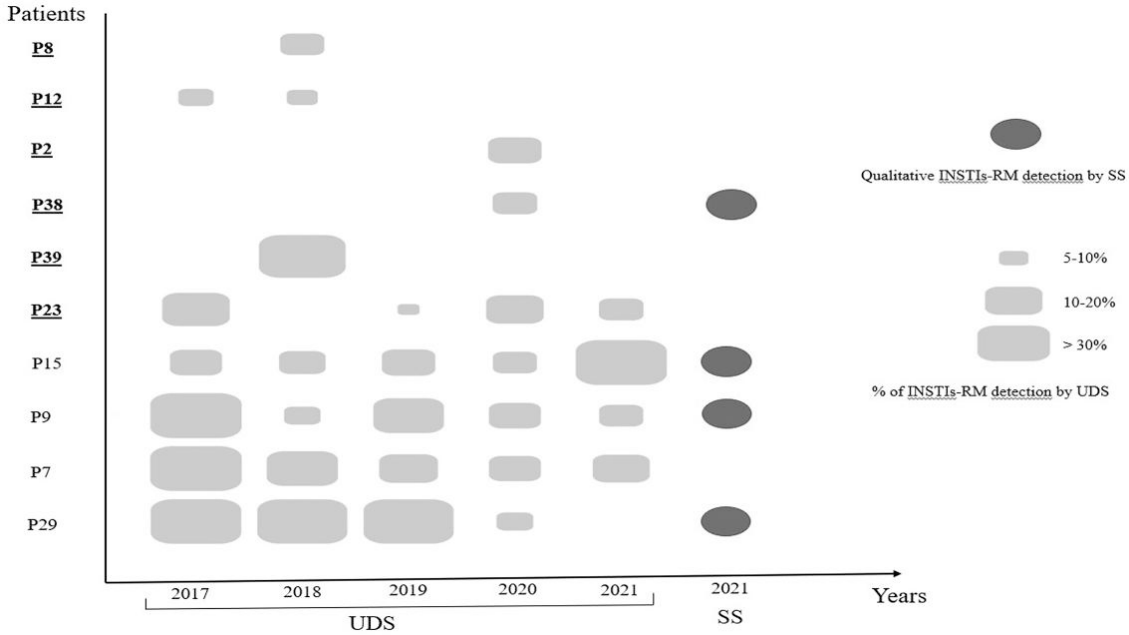
INSTI-RMs detected by SS and UDS in 10 patients. Patients in whom INSTIs-RMs were detected intermittently are indicated in bold and underlined. The empty spaces correspond to the absence of detection of INSTI-RM, either by SS or UDS. P, patient.

The INSTIs-RMs reported in the past were not detected in DNA from the blood cells of 35 of the 39 (90%) PWH by SS in 2021 (Figure 1). In the retrospective longitudinal analysis (2017–21) performed by UDS on these 35 participants, the previous INSTIs-RMs were detected in 6 of these 35 participants, with no RM detected in the other 29 (83%). Thus, the previous INSTIs-RMs seem to have been cleared in 29 of the 39 (74%) participants. The remaining 10 participants (26%) still had some or all of the INSTIs-RM previously detected. For 6 of these 10 participants, the INSTIs-RMs were detected intermittently by UDS during the analysis period (Figure 2). No additional RMs not previously detected were identified by either SS or UDS during the analysis.

### Factors associated with INSTIs-RM persistence

A comparison of immunological, virological and clinical data between the two groups (Table 1) showed that participants with persistent INSTIs-RM (INSTIs-RM^+^ group) had a higher pre-therapeutic HIV-RNA level (*P *= 0.038), lower levels of CD4 cells (*P *= 0.036) and a longer duration (*P *= 0.038) and higher level of plasma HIV-RNA replication (*P *= 0.007) on INSTIs than the INSTI-RM^−^ group. Time since HIV diagnosis, time on ART, HIV subtype and time since the last detection of INSTIs-RM did not differ between the two groups.

Univariate and multivariate logistic regression analyses were performed to assess independent associations between immunological or virological findings and the persistence of INSTIs-RM (Table 2). Mean HIV-RNA level during viral replication on INSTIs and the duration of this replication were associated with the persistence of INSTIs-RM in univariate analysis (*P *= 0.027 and 0.041, respectively). These factors remained significant in multivariate analysis, with odds ratios of 8.26 per log_10_ copies/mL (95% CI, 1.46–46.59; *P *= 0.017) and 1.05 per week of replication (95% CI, 1.00–1.11; *P *= 0.024).

**Table 2.** Univariable and multivariable analyses to identify immunological and virological factors associated with the persistence of INSTI-RMs

Characteristics	Univariable OR (95%) *P* value	Multivariable OR (95%) *P* value
Zenith HIV-RNA VL, log_10_ copies/mL	1.00 (1.00–1.00) 0.943	
CD4 nadir, cells/mm^3^	1.00 (0.99–1.03) 0.205	
Last count of CD4 cells/mm^3^	0.99 (0.99–1.00) 0.344	
Mean HIV-RNA levels during viral replication on INSTIs, log_10_ copies/mL	14.97 (1.35–165.53) 0.027	8.26 (1.46–46.59) **0.017**
Duration of viral replication on INSTIs (weeks)	1.07 (1.00–1.16) 0.041	1.05 (1.00–1.11) **0.024**

OR, odds ratio.

Univariable and multivariable models were used to identify immunological and virological factors associated with the persistence of INSTI-RMs over time. Variables with a *P* value <0.2 in univariable analysis were retained in the multivariable analysis. Statistical significance (*P *< 0.05) is indicated by the *P* values in bold.

### Ongoing ART and INSTI selection pressure

We investigated ongoing ART to determine whether participants with past detections of INSTIs-RM were being treated with INSTI-based ART and whether the selective pressure of treatment had an impact on the persistence of the MRVs concerned (Table 3).

**Table 3.** Characteristics of individuals with ongoing INSTI resistance containing regimens

Patients	INSTIs-RM previously detected in HIV-RNA genotypes	Current ART	Dose	Duration of current ART (weeks)	Time between the last detection of INSTIs-RM and INSTI treatment (weeks)
INSTIs-RM^+^					
P2	N155H	DTG + TDF + ATV	DTG double dose	80.47	4.43
P8	N155H	DTG + ATV	DTG once a day	60.17	44.23
P9	N155H	DTG + DOR + TDF + 3TC	DTG double dose	28.67	165.40
P23	G140C/S + Q148K/R	BIC + TAF + FTC + ATV	BIC once a day	41.17	111.60
P29	G140C/S + Q148K/R	DTG + DRV/r + DOR + FTR	DTG double dose	21.63	68.60
P39	G140C/S + Q148K/R	DTG + DRV/r + FTR + ENF	DTG double dose	16.73	72.57
INSTIs-RM^−^					
P3	N155H	DTG + ABC + 3TC	DTG double dose	99.77	32.13
P5	N155H	DTG + RPV	DTG double dose	51.63	34.80
P18	N155H	DTG + DOR	DTG once a day (4D)	44.23	64.30
P30	N155H	DTG + RPV	DTG once a day	48.63	55.50
P31	N155H	BIC + TAF + FTC	BIC once a day	51.93	83.80
P34	N155H	DTG + DRV/r	DTG double dose	3.97	71.87
P35	N155H	DTG + RPV	DTG once a day (5D)	41.17	105.70
P37	N155H	DTG + RPV	DTG double dose (5D)	35.60	40.23
P42	N155H + Q148K/R	DTG + TDF + FTC	DTG double dose	28.87	58.80
P14	Q148K/R	BIC + TAF + FTC	BIC once a day	45.33	61.47
P25	Q148K/R	DTG + DRV/r	DTG double dose	24.37	3.23
P41	G140C/S + Q148K/R	DTG + RPV + TAF + FTC	DTG double dose	26.27	61.30
P27	E138K + Q148K/R	BIC + TAF + FTC	BIC once a day	25.53	68.57

INSTIs-RM^+^, INSTIs-RM detected by SS or by UDS (MRV ≥ 5%); INSTIs-RM^−^, no INSTIs-RM detected by SS or UDS; DTG, dolutegravir; TDF, tenofovir disoproxil; TAF, tenofovir alafenamide; ATV, atazanavir; DOR, doravirine; 3TC, lamivudine; DRV, darunavir; r, ritonavir (as a booster); FTR, fostemsavir; ENF, enfuvirtide; ABC, abacavir.

Nineteen of the 39 participants (48.72%) were on treatments including INSTIs, and 13 of these 19 (68.42%) participants were in the INSTIs-RM^−^ group. The mutations previously detected in these participants were as follows: N155H (*n *= 8), N155H + Q148K/R (*n *= 1), Q148K/R (*n *= 2), Q148K/R + G140C/S (*n *= 1) and Q148K/R + E138K (*n *= 1). Seven of these 13 participants were on DTG-based dual therapies with rilpivirine (RPV; *n *= 4), darunavir/ritonavir (DRV/r; *n *= 2) and doravirine (*n *= 1).

## Discussion

In the context of the current expansion of the use of second-generation INSTIs in first-line ART, treatment-experienced PWH and PrEP, there is a growing need for routine clinical care studies to evaluate the feasibility of recycling these molecules in cases of prior VF on INSTI-containing regimens and INSTIs-RM selection and to identify the factors associated with the non-detectability or clearance of these RM in PBMCs.

To our knowledge, this is the first longitudinal study to have assessed the kinetics of drug-resistant variants harbouring INSTIs-RM in treatment-experienced PWH virologically controlled HIV infection and a history of VF on INSTI-containing regimens. We found that INSTIs-RMs were no longer detected by SS in PBMCs in 90% of participants. In the longitudinal UDS analysis (2017–21), the clearance of these mutations was observed in 74% of participants tested over a period of 5 years. The discrepancy between the results for the two sequencing techniques can be explained by the lower sensitivity of SS for the detection of resistant variants within quasi-species (∼20%), whereas UDS has a high sensitivity in this context.^31^

We used mutation-detection thresholds of 5%, as recommended in clinical settings, to reduce the risk of experimental artefacts and to increase reproducibility.^32^ However, UDS also has several limitations, which can explain the fluctuating or dissociated nature of RM detection in 6/10 PWH. In the setting of long-term virological suppression used here, only small amounts of HIV DNA may be present in the PBMCs DNA used as the input for the sequencing test, due to the slow turnover of the HIV DNA reservoir in cells,^33,34^ which can render RM detection stochastic. Thus, the main limitation is the likelihood of selecting a small part of the circulating reservoir, leading to insufficient drug-resistant variants capture due to dilution among a large set of mostly wild-type viral variants integrated into PBMCs. In addition, different HIV DNA proviruses have different distributions within the CD4 memory T-cell subsets, and their reactivation may depend on the immune context at the time of sampling. UDS should therefore be repeated at different time points, to confirm the clearance of RM from PBMCs. DNA genotype testing, performed in duplicate or triplicate, could also increase sensitivity but is expensive and time-consuming.^35^

Our results are consistent with those of previous studies reporting a progressive decline in RM detection rates in PWH with strict viral control.^28–30^ Nouchi *et al*.^29^ investigated the persistence and dynamics of decay of HIV-1 variants harbouring nucleoside analog reverse-transcriptase inhibitors (NRTI)- and non-nucleoside reverse transcriptase inhibitors (NNRTI) resistance mutations. They concluded that the RMs analysed were no longer detectable by UDS after 5 years in 72% of PWH. Gantner *et al*.^30^ also reported the clearance of some reverse transcriptase and protease RMs over time in PWH who had experienced significant VF in the past. In another study of the kinetics of archived M184V mutations in treatment-experienced PWH, Palich *et al*.^28^ showed that this mutation was absent in 33% of the PWH tested and that the proportion of M184V^+^ viral variants decreased significantly between 2016 and 2019 (40% versus 14%, *P *= 0.005).

As observed here for the persistence of INSTIs-RM, Palich *et al.*^28^ suggested that the persistence of the M184V mutation was associated with the duration of viral replication and the level of HIV-RNA while taking 3TC or emtricitabine (FTC). This result may be explained by the sustained seeding of the HIV reservoir with proviruses carrying RM during VF and confirms the findings of Verhofstede *et al*.^36^ who reported a correlation between the proportion of resistant variants and the time over which these variants had been able to replicate.

One of the key findings of our study is that almost half the PWH included are currently on INSTI-based regimens. Importantly, INSTIs-RM had been cleared from the blood in 13 of these PWH, despite the selective pressure exerted by DTG or BIC. We observed no emergence of other INSTIs-RM, suggesting that it should be possible to repurpose or to recycle certain antiretroviral molecules. Indeed, our results highlight the possibility of repurposing second-generation INSTIs in PWH who experienced VF for a long-acting PrEP strategy with CAB, provided that the clearance of RM from the HIV reservoir is monitored.

As the main reservoir of archived DNA RM is located in tissues, such as gut-associated lymphoid tissues, further virological investigations are important to characterize the dynamics of RM and to specify appropriate profiles of PWH with INSTIs-RM clearance for inclusion in clinical trials to assess the efficacy of recycling INSTIs in PWH with previous VF on INSTIs.

Furthermore, an analysis of INSTIs-RM dynamics in non-circulating HIV reservoirs would also be of interest, as viral genetic diversity and mutational resistance patterns can vary in some body compartments (lymph nodes and gut).^26,37^

### Conclusion

Our findings show that a clearance of archived INSTIs-RM from peripheral cell–associated HIV-1 DNA can be observed in PWH after a long period of virological control. These results provide support for the clinical evaluation of ART recycling in PWH with sustained virological control with a history of resistance and apparent clearance of archived DNA resistance from PBMCs according to repeated UDS.

## Supplementary Material

dlae194_Supplementary_Data
