# Supplementary material for: Clearance of archived integrase strand transfer inhibitors resistance mutations in people with virologically suppressed HIV infection
Source: JAC Antimicrob Resist. 2024 Dec 5;6(6):dlae194. doi: 10.1093/jacamr/dlae194 (PMC11630525; doi:10.1093/jacamr/dlae194)
Supplement: dlae194_Supplementary_Data [file dlae194_supplementary_data.docx]

**Supplementary Data**

We deep-sequenced the integrase region as two fragments: INT1and INT2.

The primers used for amplification are listed below.

**1^st^ fragment (INT1):**

**IN1-nest –for: 5’-AAGACTCGGCAGCATCTCCACTGCCACCTRTAGTAGCAAAAG-3’**

**IN1-nest -rev:**

**5’GCGATCGTCACTGTTCTCCAAGCYTGATCTCTTACCTGTCCTAT-3’**

**IN1-1er-for: 5’-GAGCAATGGCTAGTGATTTTAA-3’**

**IN1-1er-rev: 5’-ATGAATACTGCCATTTGTACTGC-3’**

**2^nd^ fragment (INT2):**

**IN2-1er-for: 5’-ACAATGGCAGCAATTTCACC-3’**

**IN2-1er-rev: 5’-TCCATGTTCTAATCCTCATCCTG-3’**

**IN2-nest-for: 5’-AAGACTCGGCAGCATCTCCAATTCCCTACAATCCCCAAAG-3’**

**IN2-nest-rev: 5’-GCGATCGTCACTGTTCTCCAAATCATCACCTGCCATCTGT-3’**

(for=forward; rev=reverse)

This gene was amplified in two rounds of PCR amplification.

PCR1 Forward (for) and PCR1 Reverse (rev) for PCR round one, and PCR 2 for and PCR2 rev for PCR round 2 (nested PCR).

The following thermocycler parameters were used:

1. **PCR1**: 50 °C for 30 minutes (min), 94 °C for 7 min, 94 °C for 10 seconds (s), 58 °C for 30 s, 68 °C for 1 min, 35 cycles of steps 3–5 and 68 °C for 7 min.

2. **PCR2**: 98 °C for 1 min, 98 °C for 10 s; 64 °C for 30 s; 72°C for 20 s, 40 cycles of steps 3-5 and 72 °C for 2 min.
